# Moving From the Old to the New: Insecticide Research on Bed Bugs since the Resurgence

**DOI:** 10.3390/insects2020210

**Published:** 2011-05-05

**Authors:** Alvaro Romero

**Affiliations:** Department of Entomology, 2310 Gardner Hall, Campus Box 7613, 100 Derieux Place, North Carolina State University Raleigh, NC 27695, USA; E-Mail: alvaro_romero@ncsu.edu; Tel.: +1-919-515-1820; Fax: +1-919-515-7746

**Keywords:** *Cimex lectularius*, resurgence, insecticides, pyrethroids, insecticide resistance, resistance mechanisms, insecticide synergism

## Abstract

The scarcity of bed bugs in many countries over the last 50 years has resulted in a lack of modern research into the toxicology of this pest. Although bed bugs resurged in the late 1990s, published research related to insecticides has lagged behind and only began to appear in 2006. The difficulty in controlling bed bugs triggered the interest of both private and academic sectors to determine the value of currently available insecticides. What follows, is updated information on effectiveness of products, studies on insecticide susceptibility, identification of mechanisms of insecticide resistance and chemical strategies proposed to overcome resistance in modern bed bug populations.

## 1. Brief History about Chemical Control of Bed Bugs

Historically, insecticides have been the principal means of controlling bed bug infestations. In the 1800s and early 1900s sprays for bed bug control were mainly based on arsenic, mercury and pyrethrum, the first two being highly toxic to humans [1,2]. These sprays were most effective against early stage infestations and direct contact of the insecticide solution with the insect was required to cause a lethal effect.

Because of the lack of residual activity of these sprays, heavy infestations very often required multiple insecticide treatments to eliminate adults or nymphs that that were missed in previous treatments [2]. A more effective way to eliminate bed bug infestations was achieved with the fumigant sulfur and later in the beginning of 20th century with hydrocyanic acid (cyanide) gas [1,2].

The discovery and wide use of dichloro-diphenyl-trichloroethane (DDT) in the 1940s changed the course of the history of bed bug control, at least in many parts of the world. The residual effect of DDT and a permissive label made this insecticide a very effective control method against bed bugs: a single and thorough application of DDT spray was generally enough to eliminate infestations and prevent re-infestations for months [2]. With DDT, direct sprays onto all individuals of an infestation were no longer necessary. Untreated insects could be killed by insects crawling on DDT-treated surfaces while searching for a host or a refuge at nighttime [2]. Wide and continued use of DDT and then later other insecticides such as malathion reduced substantially the incidence of bed bugs during the post-World War II period [1,2]. Soon after, however, several studies reported resistance of bed bugs populations to these compounds [3–5]. Despite the evidence of developing resistance, bed bug infestations continued to decrease dramatically in the USA and other developed countries over the next 40 years. Sporadic infestations in the USA occurring in dwellings with high occupant turnover and questionable sanitation conditions were eliminated with organophosphates and carbamate insecticides, none of which are available today [2,6]. Control of bed bugs today is primarily based on intensive application of a limited number of insecticides, mainly pyrethroids [7–9].

## 2. The Difficulties in Eliminating Modern Bed Bugs

In the early stages of the resurgence, a growing concern was expressed by the pest control industry because of poor effectiveness of insecticide treatments against bed bugs. A survey among U.S. pest control operators reflected these difficulties in controlling bed bugs: nearly 80% of responders indicated that to get bed bug infestations under control at least three insecticide treatments often were required, especially in cluttered environments [10]. The question that arises from such a survey was whether the difficulty in controlling bed bug infestations was due to lack of insecticide efficacy because of resistance or due to the difficulty in detecting and treating all individuals of the target population.

## 3. Initial Insecticide Studies

A first study on effectiveness of insecticides against bed bugs was conducted by Moore and Miller in 2006 [11] who performed laboratory evaluations of four pyrethroids and a chlorfenapyr based-product. They continuously exposed a laboratory susceptible strain (Fort Dix) and a field collected population to dry residues of insecticides on hardboard panels. The susceptible strain had been collected 30 years earlier and never been exposed to insecticides. Results of LT_50_s from the susceptible strain indicated that pyrethroids killed much faster (LT_50_s < 2 hours) than chlorfenapyr (LT_50_ > 9 days). A much longer time to reach 50% mortality of the field-collected population (LT_50_ > 14 days) with a deltamethin-based product, suggested the presence of pyrethroid resistance in modern bed bugs [11].

Resistance to pyrethroids was confirmed by Romero *et al.* [12] in bed bug samples collected across the USA. In this study, dose-response analysis of mortality of four field-collected bed bug strains showed a dramatic difference in susceptibility to deltamethrin when compared with results from Fort Dix strain. For these strains, the highest concentration evaluated killed less than 5% of the individuals with resistance ratio (RR) relative to the Fort Dix colony >12,765. The F_1_ offspring of matings of a highly resistant pyrethroid strain CIN1 and the susceptible Fort Dix strain showed intermediate levels of resistance (RR = 1,481). This result suggested that the genetic basis of resistance was not a single dominant-recessive gene, but it was influenced by one or more genes with incomplete dominance. The results with lambda-cyhalothrin paralleled those with deltamethrin. The CIN1 strain showed only 21.6% mortality at the highest concentration tested. Therefore, the resistance ratio was at least 6,123 [12]. Using a discriminating dose equivalent to 10 times the maximum labeled rate of deltamethrin (0.6%), 14 of 16 populations collected in Kentucky, Ohio, Michigan, New York, Massachusetts, Virginia, Florida, and California were categorized as resistant [12,13]. Detection of bed bugs resistant to pyrethroids in Denmark, the United Kingdom, and Australia indicates that pyrethroid resistance is becoming a global phenomenon [8,14,15]. Gradual loss of organophosphates and/or carbamates in countries where their use is still allowed and a greater dependence on pyrethroid insecticides to control bed bugs, will most likely increase the incidence of insecticide resistance worldwide.

## 4. Elucidating Pyrethroid Resistance Mechanisms

Since pyrethroid resistance was reported in bed bugs, several research groups have conducted studies to elucidate the resistance mechanisms involved. Initial insights on these mechanisms were provided by Yoon *et al.* [16] by sequencing DNA of voltage-gated sodium channel α-subunit gene from bed bugs collected in New York. Two mutations were identified, the Valine to Leucine mutation (V419L) and the Leucine to Isoleucine mutation (L925I). Because this population was 264-fold more resistant to 1% deltamethrin in contact bioassay, when compared with a susceptible population, the two identified mutations were likely to be responsible for deltamethrin-resistance through a knockdown-type nerve insensitivity mechanism. Further analysis of this population showed no detectable differences in the metabolic activity of glutathione transferases, esterases, and 7-ethoxycoumarin *O*-deethylases [16]. However, these results do not exclude the possibility that deltamethrin is metabolized by increased activity of one or more specific cytochrome P450 monooxygenases (P450s). Synergist studies using the cytochrome P450 monooxygenase inhibitor piperonyl butoxide (PBO) indicated that P450s contribute to, but are not wholly responsible for, deltamethrin resistance in bed bugs [17]. Transcript analysis of bed bugs exposed to pesticides showed overexpression of mRNA levels of a cytochrome P450 that has been shown to be involved in metabolic detoxication of insecticides in other arthropods [18].

Several efforts have been made to develop molecular methods for monitoring pyrethroid resistance in field bed bug populations. Seong *et al.* [19] proposed a method that identifies resistance-associated mutations which unlike conventional insecticide bioassays would require fewer individuals for a rapid diagnosis of insecticide resistance in field samples. In another study, Zhu *et al.* [20] analyzed the DNA of 100 bed bug samples collected across 17 states in the USA to determine the distribution and frequency of V419L and L925I mutations. Seventeen of those samples were further tested in residual bioassays to identify possible association between the identified mutations and resistance phenotype to deltamethrin. Results from bioassays showed deltamethrin susceptibility in only three strains while the remaining 14 samples showed moderate to high levels of resistance. Results of sequencing showed that neither of the two mutations was present in the strains susceptible to deltamethrin. Interestingly, the highly resistant pyrethroid strain CIN1, showed neither V419L nor L925I mutation in the voltage-gated sodium channel α-subunit gene which suggested that other resistance mechanisms may be responsible for deltamethrin resistance. Screening for these mutations in 93 additional bed bug samples showed that 12 contained neither of the two mutations (haplotype A) and 81 contained L925I or V419L or both mutations (haplotypes B-D) [20]. High incidence of *kdr*-like insensitivity in bed bug populations in the USA limits the effectiveness of pyrethroids and requires the selection of an insecticide that functions through a target site other than the sodium channel protein for effective control.

## 5. Coping with Insecticide Resistance

Inability to control pyrethroid resistant bed bugs necessitates development of products with new modes of action and/or the optimization of the currently available insecticides, relabeling of existing efficacious products, use of insecticide synergists, and greater reliance on alternative tactics such as heat treatment, vacuuming, mattress encasements, or barriers [21,22]. A description of published information on chemical strategies proposed to overcome insecticide resistance in bed bugs is below.

### 5.1. Insecticide Synergists

Synergism with PBO has been proposed to increase the efficacy of pyrethroids against bed bug populations that are difficult to control [23]. To investigate the potential of PBO to improve the efficacy of insecticide treatments, Romero *et al.* [17] evaluated in the laboratory the effect of the addition of commercial formulations of PBO (Exponent®) or PBO-synergized pyrethrins (Kicker®) to deltamethrin (Suspend SC®). Lack of enhancement of mortality with dry residues of deltamethrin by PBO against pyrethroid resistant strains, indicated that addition of PBO, either alone or in combination with pyrethrins, was not a comprehensive solution to deltamethrin resistance [17].

### 5.2. Dust Formulations

Little has been reported on the efficacy of dust formulations against bed bugs. Romero *et al.* [24] evaluated two pyrethroid-based dusts, DeltaDust® (0.05% deltamethrin) and Tempo 1% Dust® (1% cyfluthrin); and three desiccant dusts, Drione® (1% pyrethrins, 10% piperonyl butoxide, 40% amorphous silica gel), MotherEarth D® (100% diatomaceous earth), and NIC 325® (99.5% limestone). While Drione® and Tempo® caused 100% mortality of bed bugs within 72 hours of continuous exposure, DeltaDust® caused significant mortality (>90%) only after one week of exposure. Results of pyrethroid-based dust assays were unexpected since the individuals used in these assays were highly resistant to pyrethroids. Explanations of these findings include enhanced uptake of pyrethroid active ingredients or mortality resulting from other ‘inert’ components of the formulation. In contrast, MotherEarth D® was slower acting and only caused 100% mortality after six days of continuous exposure. Similar rates of mortality of adults exposed continuously to diatomaceous earth were found by Doggett and Russell [7]. Mortality was notably lower with limestone-based NIC 325 on all populations tested and did not exceed 50%, even after 13 days of continuous exposure [24].

### 5.3. Non-Pyrethroid Insecticides

Insect growth regulators (IGRs) such as hydroprene and methoprene, are potential alternatives to pyrethroids for managing bed bugs. Laboratory results showed that dry residues of IGRs can cause production of infertile adults, morphological malformations, incomplete ecdysis and supernumerary nymphs in individuals treated as nymphs [25,26]. However, IGRs are slow-acting insecticides on bed bugs and are generally used by the pest control industry in conjunction with other effective fast-acting insecticides [13,27].

Structural fumigation with sulfuryl fluoride is very effective against all life stages of bed bugs including eggs but requires special equipment, professional training and is considered prohibitively expensive [21,28].

Chlorfenapyr is an option that is registered for bed bug control and is increasingly being used commercially [27,29,30]. Laboratory evaluations with two chlorfenapyr-based formulations (Phantom SC® and Phantom aerosol®) indicated that chlorfenapyr kills bed bugs as both a contact spray and dry residue. In dry residue assays with Phantom SC, mortality of pyrethroid resistant bed bugs >90% was recorded after five days of continuous exposure [31]. Moore and Miller [11] reported a much longer exposure time of bed bugs to hardboard panels treated with this insecticide to obtain 50% mortality. Phantom aerosol® caused mortality about 1.5 and 3 times faster, respectively, than Phantom SC®. The difference in mortality rate between the two formulations could be due to greater bioavailability of the active ingredient or synergism with other ingredients in the aerosol formulation. Under laboratory conditions, dry residues of Phantom SC aged for four months on filter paper were as toxic as fresh dry residues [31]. The ability of chlorfenapyr to remain effective over an extended period of time is encouraging because bed bugs that are not sprayed directly may still succumb after residing on treated surfaces. Further study is warranted on the longevity and availability of chlorfenapyr on wood, fabric and similar substrates commonly occupied by bed bugs.

## 6. Conclusions

A number of insecticide studies have been published in the last decade, including studies on efficacy of insecticides, resistance, and synergists. Initial laboratory evaluations with field collected strains demonstrated a high prevalence of pyrethroid resistance in bed bug populations. Detection of resistance is not surprising as bed bugs have a history of developing resistance to insecticides. However, it is intriguing to see that the majority of bed bug populations screened in the USA are resistant to pyrethroids despite the absence of treatments over 50 years. One hypothesis for explaining this is that resistance alleles already may have been present in populations because of cross resistance between DDT and pyrethroids [13]. The difficulty in eliminating resistant bed bugs has renewed interest in developing effective control tactics. The synergist PBO emerged as an option to enhance toxicity of pyrethroids. Synergism studies and the identification of mutations associated to a knockdown-type nerve insensitivity mechanism indicated that PBO might only be effective against pyrethroid-resistant bed bugs in which P450s are primarily responsible for the detoxification of the insecticide. The presence of target site insensitivity may be countered by selection of insecticides with different modes of action. Chlorfenapyr is one option, although studies showed that it is relatively slow acting. Insect growth regulators require more laboratory and field studies to determine their potential for bed bug control. Dust formulations containing silica gel or diatomaceous earth kill pyrethroid-resistant bed bugs, but the utility of dusts is limited by where they can be legally or prudently applied. Insecticides will continue to play a significant role in bed bug control. However, the number of options for chemical control of bed bugs has decreased due to the regulatory restriction of effective insecticides in many countries. There are a number of U.S. Environmental Protection Agency EPA exempt botanical insecticides that are available for bed bug management but their effectiveness remains to be investigated. The future for bed bug control might well depend on the availability of potent and safe dry residual insecticides and the incorporation of alternative tactics in the management programs.
